# Effects of Tannic Acid Supplementation on the Intestinal Health, Immunity, and Antioxidant Function of Broilers Challenged with Necrotic Enteritis

**DOI:** 10.3390/antiox12071476

**Published:** 2023-07-24

**Authors:** Huiping Xu, Xiaodan Zhang, Peng Li, Yimeng Luo, Jianyang Fu, Lu Gong, Zengpeng Lv, Yuming Guo

**Affiliations:** 1 State Key Laboratory of Animal Nutrition, College of Animal Science and Technology, China Agricultural University, Beijing 100193, China; 2 Engineering Research Center of Feed Protein Resources on Agricultural By-Products, Ministry of Education, Hubei Key Laboratory of Animal Nutrition and Feed Science, Wuhan Polytechnic University, Wuhan 430023, China

**Keywords:** anti-inflammatory, antioxidant capacity, broiler, intestinal health, necrotic enteritis, tannic acid

## Abstract

*Clostridium perfringens* causes necrotic enteritis (NE) after proliferation in the intestine of poultry, resulting in considerable losses to the poultry industry. This study aimed to investigate the impact of tannic acid on the antioxidant, immunity, and gut health of broilers with NE. In the experiment, 630 one-day-old Cobb500 male chicks were randomly divided into six treatment groups, with seven replicate cages and with fifteen birds in each cage. The treatment groups were as follows: control group (NC), challenged group (PC), and challenged NE chickens treated with 250, 500, 750, and 1000 mg/kg tannic acid (PTA1, PTA2, PTA3, and PTA4, respectively). To induce NE, coccidia vaccine and *Clostridium perfringens* were administered on day 19 and days 22–28, respectively. Indexes related to antioxidant, immune, and intestinal health were measured on days 28 and 35. During the infection period, we observed significant increases in fecal water content, D-LA, TNF-α, and malondialdehyde concentrations (*p* < 0.05). Conversely, significant decreases were noted in chyme pH and in T-AOC, IL-4, and IL-10 concentrations (*p* < 0.05). The addition of tannic acid exhibited a linear decrease in fecal water content and TNF-α concentration (*p* < 0.05). Furthermore, tannic acid supplementation resulted in a quadratic curve decrease in D-LA concentration and linear increases in T-AOC, IL-4, and IL-10 (*p* < 0.05). Cecal microbiological analysis revealed that Ruminococcaceae and *Butyricimona* were dominant in PTA3. In conclusion, the dietary addition of tannic acid may reduce the negative effects of NE by increasing antioxidant and anti-inflammatory capacity, improving the intestinal barrier, and regulating the intestinal flora.

## 1. Introduction

*Clostridium perfringens* (*C. perfringens*) can decompose nutrients and produce large amounts of gas, resulting in tissue aerocele and extensive necrosis. It is the main pathogen that causes necrotic enteritis (NE) in poultry [1]. NE, known as enterotoxemia, is mainly caused by *C. perfringens* type A, which often accompanies or occurs successively with coccidia in production, considerably impacting the economy of the poultry breeding industry, with an estimated annual loss of up to USD 6 billion [2,3,4]. Antibiotic growth promoters (AGPs) can be used to protect broilers against intestinal diseases and improve their productivity. In light of the emergence of bacterial resistance and concerns about antibiotic residues in animal products, however, numerous countries and regions have implemented restrictions and bans on the use of AGPs in livestock and poultry feeds. Consequently, there is a pressing need for research investigating suitable alternatives to AGPs to effectively manage the rising occurrence of NE in chickens.

A range of polyphenols are present in plants. Among them, tannic acid, a secondary metabolite produced by plants, has potential as a feed additive [5]. In monogastric animals, supplementation of different concentrations of tannins has had positive effects owing to their antioxidant, antimicrobial, and anti-inflammatory properties [6,7,8]. Studies have shown that the addition of tannic acid to diets reduces the colonization of intestinal pathogenic bacteria, including *Escherichia coli* and *Salmonella*, and promotes intestinal health [9,10]. In vitro studies have reported that tannic acid can inhibit the reproduction of *C. perfringens* [11,12]. Although there has been progress in the application of tannic acid in broiler feed, its effects on the intestinal health and antioxidant function of broilers with NE have not been fully elucidated. Several investigations have examined alterations in cytokine concentrations in broilers afflicted with NE [13,14,15]. However, there is a need for more robust and dependable data regarding the immunomodulatory impacts of tannins on NE-infected broilers. Therefore, comprehending the effects of tannins on intestinal health, immune response, and antioxidant activity in NE-infected broilers is a crucial effort due to the promise tannins hold as an alternative to antibiotics.

In this study, we have investigated the effects of adding different concentrations of tannic acid on the fecal water content, intestinal barrier integrity, chyme pH, intestinal and serum antioxidant capacity and immunity, and intestinal microorganisms in broilers infected with NE. This study was conducted to verify whether the addition of appropriate doses of tannic acid could reduce the negative effects of NE by increasing the antioxidant capacity and improving the intestinal health of broilers, and provides new scientific data for the application of tannic acid in broilers.

## 2. Materials and Methods

### 2.1. Experimental Design

The experiment was conducted at the poultry experiment base of China Agricultural University, located in Zhuozhou City, China. In the experiment, 630 one-day-old Cobb500 male chicks were randomly divided into six treatment groups, with seven replicate cages and fifteen birds in each cage. Broilers were housed in two-tiered battery cage units, with the battery cage as the experimental unit. The treatment groups were as follows: negative control group (NC), unchallenged and untreated; positive control group (PC), challenged with NE and untreated; PTA1, challenged with NE and administered control diet supplemented with 250 mg/kg tannic acid; PTA2, challenged with NE and administered control diet supplemented with 500 mg/kg tannic acid; PTA3, challenged with NE and administered control diet supplemented with 750 mg/kg tannic acid; and PTA4, challenged with NE and administered control diet supplemented with 1000 mg/kg tannic acid. Broilers were given ad libitum access to feed and water during the experiment. All chickens were vaccinated and subjected to standard immunization and management protocols for Cobb broilers. The test diets were in pelleted form with reference to the Chinese Chicken Feeding Standard (NY/T 33-2004) (http://down.foodmate.net/standard/yulan.php?itemid=7410 (accessed on 20 June 2023)) and were formulated as a corn-soybean meal-based diet. The formulation of the basic diet is shown in Table 1.

### 2.2. Challenging Broilers with Necrotic Enteritis

**Figure 1:**
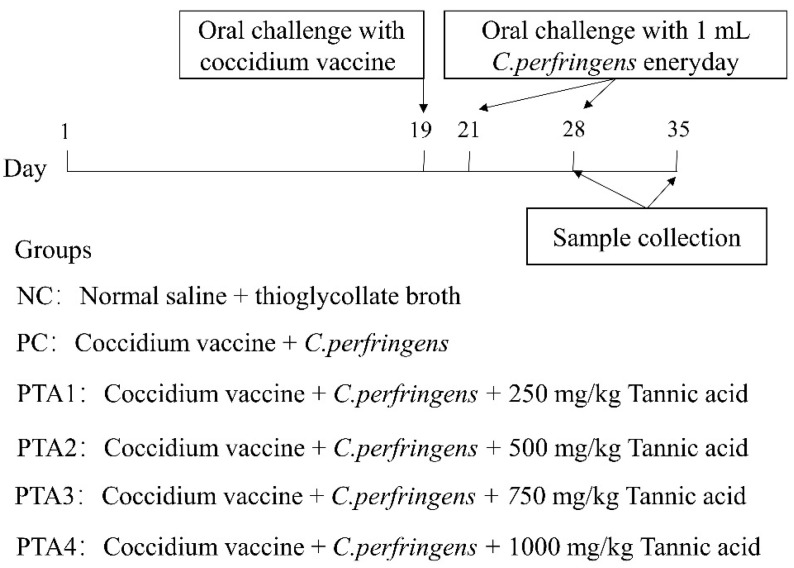
Flowchart of the animal experiment. The type of *C. perfringens* in this test was chicken-derived *C. perfringens* type A CVCC52.

Broilers were challenged with NE, as described in a previous study (co-infection of broilers with coccidia and *C. perfringens*) with slight modifications [16]. Compared with previous studies, we adjusted the dose and duration of infection for *C. perfringens* and coccidia in the current study accordingly. The process employed to establish the NE model is illustrated in Figure 1. With the exception of the NC group, all groups were administered a 1 mL oral dose of attenuated quadrivalent coccidia vaccine suspension (Foshan Zhengdian Biotechnology Co., Ltd., Foshan, China) at a 25-fold dose on d 19. The NC group received an equivalent volume of saline via oral gavage. During d 22–28, the chickens in the NC group were gavaged 1 mL of sterile fluid medium, whereas the birds in the other groups were gavaged 1 mL of *C. perfringens* type A CVCC52 (3 × 10^8^ CFU/mL).

### 2.3. Fecal Water Content

The 24 h fecal samples of broilers were collected on d 25, 26, 27, 28, and 35 of the experiment; approximately 400 g of feces was collected after kneading, and was weighed, and dried in an oven at 65 °C for 3 days. The weight was recorded after constant weight and the initial water content of feces was calculated. The average value of the water content in d 25–28 manure was calculated to represent the initial water content of manure at d 25–28. The initial water content of fecal samples (%) was calculated as follows: The initial water content of fecal samples = (fresh weight of fecal samples − dry weight of fecal samples)/fresh weight of fecal samples × 100%.

### 2.4. Determination of Ileum and Cecum Chyme pH

Chyme pH was determined according to the method described by Burnell et al. [17]. In brief, the entire contents of the ileum and cecum were collected and thawed on ice. Then, approximately 1.5 g of chyme was added to a 15 mL centrifuge tube and diluted with deionized water at a ratio of 1:8 (*w*:*v*). The solution was shaken and mixed with a vortex shaker for 10 min. Finally, the pH was measured using a pH meter (METTLER TOLEDO, Shanghai, China).

### 2.5. Determination of Serum Diamine Oxidase and D-Lactic Acid Concentrations

The concentrations of diamine oxidase (DAO) and D-lactic acid (D-LA) in blood were measured using the DAO kit (A088-1, Nanjing Jiancheng Bioengineering Institute, Nanjing, China) and D-LA kit (E-BC-K002-M, Wu Han Elabscience Biotechnology Co., Ltd., Wuhan, China) according to the manufacturer’s instructions.

### 2.6. Determination of Cytokines in Ileum and Serum

A 0.1 g sample of middle ileum tissue was collected and homogenized with ten times the volume of pre-cooled PBS. The supernatant was then subjected to thorough homogenization and the protein content was subsequently measured. The concentrations of TNF-α (ml002790, Shanghai Enzyme-linked Biotechnology Co., Ltd., Shanghai, China), IL-4 (ml059838, Shanghai Enzyme-linked Biotechnology Co., Ltd., Shanghai, China), and IL-10 (ml059830, Shanghai Enzyme-linked Biotechnology Co., Ltd., Shanghai, China) in the ileal tissue homogenate and serum were determined using enzyme-linked immunoassay kits. The determination procedures were conducted following the instructions provided by the manufacturer.

### 2.7. Determination of Antioxidant Indexes in Serum and Ileal Mucosa

Blood collected from the wing vein was centrifuged at 1500× *g* for 10 min at 4 °C to separate the serum, which was stored at −80 °C. The total antioxidant capacity (T-AOC), malondialdehyde (MDA) content, and superoxide dismutase (SOD) activity were measured using the collected serum. MDA (A003-1, Nanjing Jiancheng Bioengineering Institute, Nanjing, China) was determined using the serum stock solution. For the determination of SOD, one tube was prepared by diluting the serum volume with a 1:19 ratio of saline volume (A001-3, Nanjing Jiancheng Bioengineering Institute, Nanjing, China). A ratio of serum volume:saline volume of 1:4 was used for determining T-AOC (A015-2-1, Nanjing Jiancheng Bioengineering Institute, Nanjing, China). The middle ileal mucosa collected on d 28 and 35 was prepared into a 10% homogenate using precooled saline, and mucosal MDA (A003-1, Nanjing Jiancheng Bioengineering Institute, Nanjing, China) and total protein (TP, A045-2-2, Nanjing Jiancheng Bioengineering Institute, Nanjing, China) were measured. Precooled normal saline was added to the homogenate and diluted according to the ratio of 10% homogenate volume:normal saline volume = 1:19 for SOD determination (A001-3, Nanjing Jiancheng Bioengineering Institute, Nanjing, China). The T-AOC (A015-2-1, Nanjing Jiancheng Bioengineering Institute, Nanjing, China) of the ileal mucosa was measured according to the ratio of 10% homogenate volume:normal saline volume = 1:4. Serum enzyme activity was expressed as U/mL and ileal mucosal enzyme activity was expressed as U/mg of protein. All assay procedures were performed according to the manufacturer’s instructions.

### 2.8. Sequencing of Cecal Microorganisms

Cecal microbial sequencing was performed according to the method described by Zhang et al. [18]. Bacterial genomic DNA was extracted from 0.2 g cecal chyme samples using the QIAamp DNA Stool Mini Kit (Qiagen Inc., Valencia, CA, USA) according to the manufacturer’s instructions. DNA was examined using 1% agarose gel electrophoresis. After dilution, the genomic DNA served as a template for amplifying bacterial DNA using the universal primers 338F (5′-ACTCCTACGGGAGGCAGCA-3′) and 806R (5′-GGACTACHVGGGTWTCTAAT-3′), targeting the V3-V4 region of the 16S rDNA gene. Subsequently, the PCR products were purified, quantified, and homogenized to create a sequencing library. The resulting libraries were assessed for quantity using Qubit and Q-PCR before being sequenced on a NovaSeq6000 platform. Sequencing procedures were conducted by Beijing Novogene Co., Ltd. (Beijing, China). The number of operational taxonomic units (OTUs), Shannon index, Simpson index, abundance-based coverage estimator index, and Chao1 index were calculated for each sample using Qiime software (Qiime2-2019.7, Nature Biotechnology, USA). Bacterial populations with differential abundance were analyzed using the linear discriminant analysis effect size (LEfSe) method.

### 2.9. Statistical Analysis

The data were analyzed using SPSS 26.0 (SPSS, Inc., Chicago, IL, USA) and compared using one-way analysis of variance (ANOVA) and Duncan’s multiple comparisons. The challenged groups (PC, PTA1, PTA2, PTA3, and PTA4) were analyzed using contrast tests for the linear and quadratic effects of different doses of tannic acid. Results are expressed as mean ± standard error of mean (SEM). A *p*-value of <0.05 was considered a significant difference. Pearson’s correlation analysis was employed to assess the relationships between the microbiota and various indicators of antioxidation, intestinal health, immunity, and chyme pH in broilers at 28 and 35 days of age.

## 3. Results

### 3.1. Fecal Water Content 

Table 2 shows the effects of tannic acid on the fecal water content of broilers infected with NE. The PC group had significantly increased fecal water content on d 28, 25–28, and 35 compared with the NC group (*p* < 0.05). The addition of tannic acid linearly decreased the fecal water content on d26, 25–28, and 35 compared with the PC group (*p* < 0.05). Tannic acid addition significantly decreased the fecal water content on d26 compared with the NC group (*p* < 0.05); however, it had no significant effect on the fecal water content on other days.

### 3.2. pH of Ileal and Cecum Chyme

The impact of tannic acid on the chyme pH of broilers afflicted with NE is presented in Table 3. On d 28, the pH of the ileal and cecum chyme in the PC group exhibited a significant decrease compared to the NC group (*p* < 0.05). However, no significant difference in the pH of ileal and cecum chyme was observed between the PC and NC groups on d 35. Further, tannic acid addition had no significant effect on the pH of ileal and cecum chyme.

### 3.3. Serum D-LA and DAO Concentrations

Table 4 shows the results of the effects of tannic acid addition on D-LA and DAO concentrations in the serum of broilers infected with NE. The serum D-LA concentrations were significantly increased in the PC group compared with the NC group on d 28 (*p* < 0.05). Although there was no statistically significant difference between the NC and PC groups in serum DAO concentrations, numerically the serum DAO concentrations in broilers in the PC group were elevated by approximately 35%. Compared with the PC group, the PTA2 and PTA3 groups showed a significant decrease in serum D-LA concentrations on d 28 (*p* < 0.05). Tannic acid addition did not have a significant effect on serum DAO concentrations on d 28; however, serum DAO concentrations were similar to that in the NC group. On d 35, serum DAO concentrations in PTA2, PTA3, and PTA4 groups decreased significantly compared with the PC group (*p* < 0.05).

### 3.4. Cytokine Contents in Serum and Ileum

Table 5 presents the impact of tannic acid on the immunity of broilers infected with NE. The results reveal that NE infection significantly elevated the concentrations of TNF-α in the serum and ileum (*p* < 0.05) while significantly reducing the concentrations of IL-4 and IL-10 (*p* < 0.05) compared to the NC group. Furthermore, the addition of tannic acid exhibited a linear decrease in TNF-α concentrations (*p* < 0.05) and a linear increase in IL-4 and IL-10 concentrations (*p* < 0.05). Specifically, regarding the cytokine concentrations in the serum, the PTA2, PTA3, and PTA4 groups showed significantly decreased serum concentrations of TNF-α (*p* < 0.05) and significantly increased serum concentrations of IL-4 and IL-10 (*p* < 0.05) compared to the PC group. In addition, PTA1 had significantly increased serum concentrations of IL-10 on d 28 and d 35 as well as IL-4 on d 35 compared to the PC group (*p* < 0.05).

In the ileal tissue, the PTA1, PTA3, and PTA4 groups had significantly decreased concentrations of TNF-α in the ileum on d 28 compared to the PC group (*p* < 0.05). Moreover, the PTA2, PTA3, and PTA4 groups had reduced concentrations of TNF-α in the ileum on d 35 (*p* < 0.05). The addition of tannic acid significantly increased the concentrations of IL-4 and IL-10 in the ileum on d 35 compared to the PC group (*p* < 0.05), though it did not have a significant effect on IL-10 in the ileum on d 28.

### 3.5. Antioxidant Function of Serum and Ileal Mucosa

Table 6 presents the effects of tannic acid on the antioxidant function of serum and ileal mucosa in broilers with NE. Serum MDA concentrations were significantly increased in the PC group compared with the NC group on d 28 (*p* < 0.05). Serum T-AOC was significantly increased in the PTA4 group compared with the PC group on d 28 (*p* < 0.05).

For ileal mucosa, the PC group had significantly decreased T-AOC compared with the NC group on d 28 (*p* < 0.05). Overall, tannic acid addition had no significant effect on the antioxidant capacity of the ileal mucosa; however, it linearly increased the T-AOC of the ileal mucosa.

### 3.6. Cecal Microorganisms

**Figure 2:**
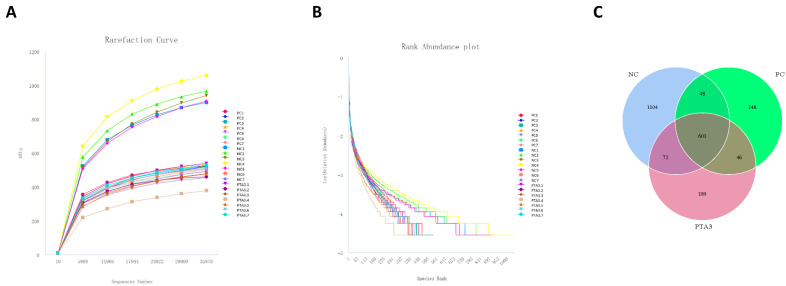
Rationalization of cecum microbial sequencing data volume and number of OTUs in the NC, PC, and PTA3 groups on d 28: (**A**) shows the rarefaction curve of cecal microorganisms, (**B**) shows the rank abundance of cecal microorganisms, and (**C**) shows the OTUs specific to cecal microorganisms in the NC, PC, and PTA3 groups. NC: unchallenged and untreated; PC: challenged with NE and untreated; PTA3: challenged with NE and treated with a control diet supplemented with 750 mg/kg tannic acid. *n =* 7.

**Figure 3:**
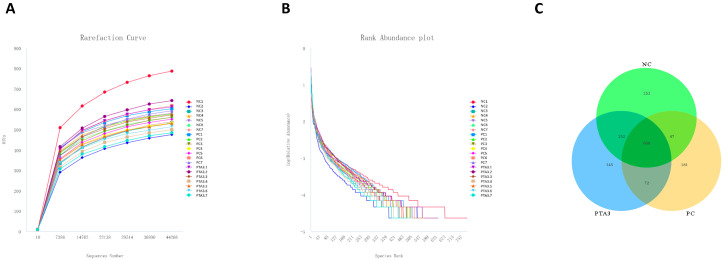
Rationalization of cecal microbial sequencing data volume and number of OTUs in the NC, PC, and PTA3 groups on d 35: (**A**) shows the rarefaction curve of cecal microorganisms, (**B**) shows the rank abundance of cecal microorganisms, and (**C**) shows the OTUs specific to cecal microorganisms in the NC, PC, and PTA3 groups. NC: unchallenged and untreated; PC: challenged with NE and untreated; PTA3: challenged with NE and treated with a control diet supplemented with 750 mg/kg tannic acid. *n =* 7.

We constructed rarefaction curves and rank abundance plots to assess whether the sequencing volume was sufficient for subsequent analysis (Figure 2A,B and Figure 3A,B). The rarefaction curves and rank abundance increased with sequencing volume and the samples approached a horizontal state, indicating that the sequencing volume could capture most of the flora composition in the samples and meet the needs of the subsequent analysis.

**Figure 4:**
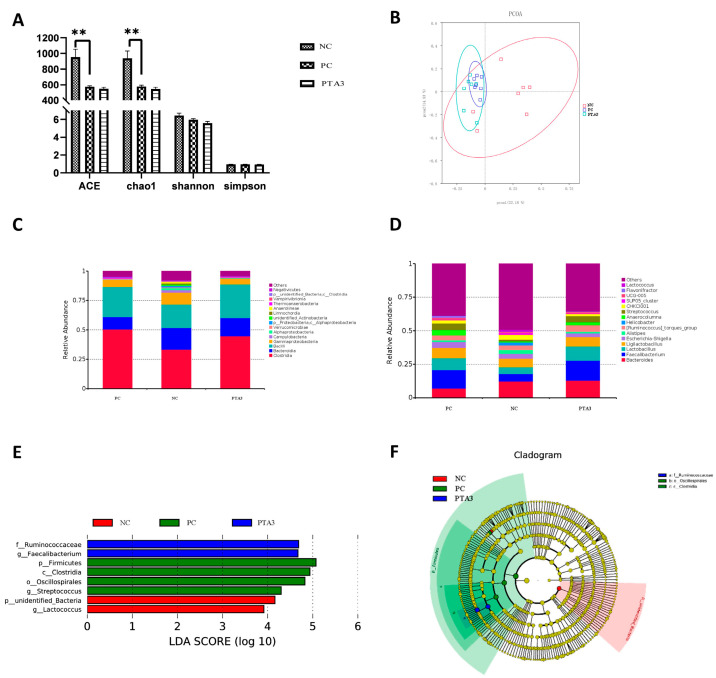
Effects of tannic acid addition on cecal microorganisms in broiler chickens with NE on d 28: (**A**) shows the effects of tannic acid on cecal microbial α-diversity; (**B**) shows the PCoA plot; (**C**) shows the relative abundance of the top fifteen microorganisms at the class level; (**D**) shows the relative abundance of the top fifteen microorganisms at the genus level; (**E**) is the result of LEfSe analysis for differential microorganisms; and (**F**) shows the Cladogram plots of the differential microorganisms. NC: unchallenged and untreated; PC: challenged with NE and untreated; PTA3: challenged with NE and treated with a control diet supplemented with 750 mg/kg tannic acid. *n =* 7. ** indicates *p* < 0.01.

On d 28, cecal microbial analysis showed the presence of 1104 specific OTUs in the NC group, 148 OTUs in the PC group, and 189 OTUs in the PTA3 group (Figure 2C). A significant decrease in α-diversity was observed between the PC and NC groups (*p* < 0.05), while no significant difference in α-diversity was observed between the PC and PTA3 groups (Figure 4A). Figure 4B shows the PCoA plot of cecal microorganisms on d 28; combined with Table 7, a significant difference in β-diversity can be observed between the NC and PC groups and between the PC and PTA3 groups (*p* < 0.05). Figure 4C,D presents the relative abundance of the top fifteen species by class and genus concentrations on d 28. LEfse analysis (Figure 4E,F) revealed that Bacteria and *Lactococcus* were dominant in the NC group, while Clostridia, Oscillospirales, and *Streptococcus* were dominant in the PC group. Ruminococcaceae and *Faecalibacterium* were more dominant in the PTA3 group.

**Figure 5:**
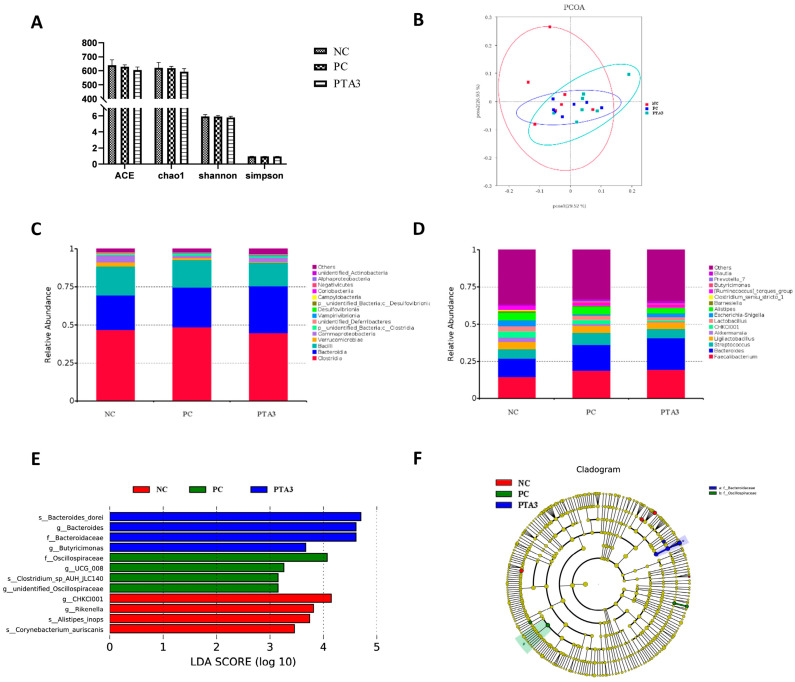
Effects of tannic acid addition on the cecal microorganisms in broiler chickens with NE on d 35: (**A**) shows the effects of tannic acid on cecal microbial α-diversity; (**B**) shows the PCoA plot; (**C**) shows the relative abundance of the top fifteen microorganisms at the class level; (**D**) shows the relative abundance of the top fifteen microorganisms at the genus level; (**E**) shows the result of the LEfSe analysis of the differential microorganisms; and (**F**) shows the Cladogram plots of the differential microorganisms. NC: unchallenged and untreated; PC: challenged with NE and untreated; PTA3: challenged with NE and treated with a control diet supplemented with 750 mg/kg tannic acid. *n =* 7.

Cecal microbial analysis on d 35 revealed 252 specific OTUs in the NC group, 181 in the PC group, and 145 in the PTA3 group (Figure 3C). No significant differences in α-diversity were observed among the three groups (Figure 5A). PCoA plots of cecal microorganisms on d 35 are presented in Figure 5B. Combined with the information provided in Table 7, a significant difference in β-diversity was found between the NC and PC groups (*p* < 0.05), and a suggestive difference in β-diversity was observed between the PC and PTA3 groups (*p* = 0.089). The relative abundance of the top fifteen species at the class and genus concentrations on d 35 is illustrated in Figure 5C,D. LEfse analysis (Figure 5E,F) revealed that the differential genera were *CHCKI001*, *Rikenella*, *alistipes_inops*, and *corynebacterium_auriscanis* in the NC group and Oscillospiraceae, *UCG_008*, and *Clostridium* sp. *AUH-JLC140* in the PC group. *Bacteroidaceae*, *Bacteroides_dorei* and *Butyricimonas* were more dominant in the PTA3 group.

### 3.7. Indicators Correlation Analysis

**Figure 6:**
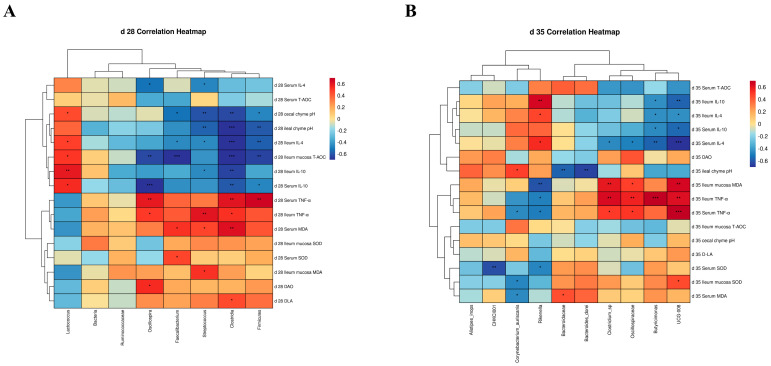
Correlation analysis of antioxidance, immunity, intestinal barrier, and chyme pH with differential bacteria: (**A**) shows the results of the correlation analysis on d 28 and (**B**) shows the results of the correlation analysis on d 35. Probability values are indicated as follows: * *p* < 0.05, ** *p* < 0.01, *** *p* < 0.001.

To explore potential correlations between intestinal flora and antioxidation, intestinal barrier function, and chyme pH, a correlation analysis was conducted. The correlation heat map for the indicators on d 28 is displayed in Figure 6A. The findings revealed several significant correlations between specific bacteria and various parameters. *Faecalibacterium* showed a significant positive correlation with serum SOD (*p* < 0.05). *Clostridia* exhibited a highly significant positive correlation with serum MDA (*p* < 0.01), while *Faecalibacterium* and *Streptococcus* showed a significant positive correlation (*p* < 0.05). *Streptococcus* displayed a positive correlation with ileal mucosal MDA (*p* < 0.05). Significant positive correlations were observed between serum D-LA and *Clostridia* (*p* < 0.05), as well as between serum DAO and Oscillospira (*p* < 0.05). Ileal mucosal T-AOC exhibited highly significant negative correlations with *Faecalibacterium* and Clostridia (*p* < 0.001), as well as highly significant negative correlations with Firmicutes and Oscillospira (*p* < 0.01). However, ileal mucosal T-AOC displayed a significant positive correlation with *Lactococcus* (*p* < 0.05). Both ileal and cecum chyme pH demonstrated significant negative correlations with Firmicutes (*p* < 0.05), Oscillospira (*p* < 0.01), *Faecalibacterium* (*p* < 0.05), and *Streptococcus* (*p* < 0.01). However, ileal chyme pH showed a significant positive correlation with *Lactococcus* (*p* < 0.05). Serum TNF-α content exhibited a significant positive correlation with Firmicutes, Clostridia, and Oscillospira (*p* < 0.01). The TNF-α content in the ileum showed positive correlations with Clostridia and Oscillospira (*p* < 0.05) and a significant positive correlation with *Streptococcus* (*p* < 0.01). Serum IL-4 content displayed a significant negative correlation with *Streptococcus* and Oscillospira (*p* < 0.05). IL-4 content in the ileum exhibited significant negative correlations with *Streptococcus*, *Faecalibacterium*, Firmicutes, and Clostridia (*p* < 0.05) while showing a significant positive correlation with *Lactococcus* (*p* < 0.05). Serum IL-10 content was significantly negatively correlated with Firmicutes (*p* < 0.05), Clostridia (*p* < 0.01), and Oscillospira (*p* < 0.001). IL-10 in the ileum demonstrated a significant negative correlation with *Streptococcus* (*p* < 0.05) and Clostridia (*p* < 0.01). Both ileal and serum IL-10 content exhibited significant positive correlations with *Lactococcus* (*p* < 0.05).

Figure 6B illustrates the correlation heat map for the indicators on d 35. The results reveal that ileal chyme pH had a highly significant negative correlation with *Bacteroides_dore* and Bacteroidaceae (*p* < 0.01) and a significant positive correlation with *Corynebacterium_auriscanis* (*p* < 0.05). The content of ileal mucosal MDA exhibited a highly significant positive correlation with *Clostridium_sp* and *UCG-008* (*p* < 0.01), a positive correlation with *Oscillospiraceae* (*p* < 0.05), and a highly significant negative correlation with *Rikenella* (*p* < 0.01). Serum SOD displayed a highly significant negative correlation with *CHKCI001* (*p* < 0.01) and *Rikenella* (*p* < 0.05). The serum MDA content showed a significant positive correlation with Bacteroidaceae (*p* < 0.05) and a significant negative correlation with *Corynebacterium_auriscanis* (*p* < 0.05). The content of ileal mucosal SOD exhibited a significant positive correlation with *UCG-008* (*p* < 0.05) and a significant negative correlation with Corynebacterium (*p* < 0.05). Serum TNF-α content was significantly positively correlated with *Clostridium_sp*, *Oscillospiraceae* (*p* < 0.05), and was extremely significantly positively correlated with *UCG-008* (*p* < 0.001). However, it was significantly negatively correlated with *Rikenella* and *Corynebacterium_auriscanis* (*p* < 0.05). TNF-α content in the ileum showed a significant positive correlation with *UCG-008*, *Clostridium_sp*, and *Oscillospiraceae* (*p* < 0.01) and an extremely significant positive correlation with *Butyricimonas* (*p* < 0.001), while there was a significant negative correlation with *Rikenella* (*p* < 0.05). IL-10 and IL-4 contents were consistent with the bacterial correlations, mainly showing a negative correlation with *UCG-008* and *Butyricimonas* (*p* < 0.05). The content of IL-4 in the serum and ileum, as well as IL-10 in the ileum, showed a significant positive correlation with *Rikenella* (*p* < 0.05). Additionally, serum IL-4 was significantly negatively correlated with *Clostridium_sp* and *Oscillospiraceae* (*p* < 0.05).

## 4. Discussion

This study aimed to investigate the effects of tannic acid on the antioxidant function, immunity and intestinal health of broiler chickens infected with NE. Oxidative stress plays an important role in NE pathogenesis [19]. NE can increase MDA concentrations in the body [20,21] and decrease the activities of antioxidant enzymes such as catalase [22]. In the present study, we observed that NE reduced the antioxidant capacity of the body by decreasing T-AOC and increasing MDA concentrations; these results are consistent with those of previous studies [23,24]. Oxidative damage is caused by an imbalance between antioxidant defense and free radical generation systems [25]. Throughout evolution, most organisms have developed innate enzymatic defense mechanisms, non-enzymatic antioxidant defenses, and repair systems to safeguard against oxidative stress-induced damage. However, these natural antioxidant systems often fall short in providing adequate protection. Hence, the search for antioxidants to counteract oxidative damage is of the utmost importance. Numerous studies have substantiated the antioxidant properties of tannic acid [26,27,28,29,30,31]. Tannins are rich in hydroxyl groups; the hydrogen released from these hydroxyl groups exhibits a strong antioxidant capacity by scavenging free radicals. Further, tannic acid may reduce oxidative stress by activating the Nrf2-Keap1 pathway [28]. However, in the current investigation tannic acid did not exhibit outstanding antioxidant capacity. Nevertheless, it dose-dependently elevated the T-AOC of both the serum and ileal mucosa, indicating a dose-dependent antioxidant effect of tannic acid. A previous study has reported that the antioxidant function of tannic acid is related to its structure and polymerization degree [32]; this could be the reason why tannic acid exhibited no prominent antioxidant capacity in the current study.

The rate of diarrhea was significantly higher in broiler chickens with intestinal bacterial infection [33,34], as demonstrated by the significant increase in fecal water content found in this study. We observed that addition of tannic acid to the diet linearly reduced fecal water content and was effective in suppressing the increase in fecal water content caused by NE; this result is consistent with that of Yang et al. [35], Girard et al. [36], and Choi et al. [37], who showed that addition of tannic acid to the diet alleviated diarrhea in animals and reduced fecal water content and the duration of diarrhea. The potential antidiarrheal effect of tannic acid can be attributed to the following factors: First, tannic acid acts as an astringent when it enters the animal’s intestine, which leads to a slowdown in intestinal peristalsis and an enhancement in water reabsorption. Second, tannic acid has the ability to improve intestinal health and enhance the intestinal barrier in chickens by promoting favorable changes in intestinal morphology and upregulating the expression of genes related to intestinal tight junction proteins. Lastly, tannic acid exhibits selective antibacterial properties, and the reduction of cytotoxin production by bacteria is one of the contributing factors to the alleviation of diarrhea symptoms in broilers. Tannic acid has inhibitory effects on pathogenic bacteria such as *C. perfringens* and *Salmonella*, thereby reducing the occurrence of diarrhea [12,38,39].

Maintaining optimal intestinal pH is important in order to maximize nutrient utilization and maintain a steady state of intestinal microbiota. Any changes in the intestinal flora may affect chyme pH. In this study, broiler chickens infected with NE exhibited a notable reduction in ileal and cecum chyme pH. Several potential factors could contribute to this observation, including alterations in intestinal flora caused by NE infection, diminished digestive capacity, and the fermentation of undigested nutrients serving as substrates for intestinal microorganisms. Intestinal pH has been found to be reduced under unfavorable conditions such as heat stress and NE [40,41,42], which is consistent with the results of the present study. Tannic acid addition did not significantly affect ileal and cecum chyme pH in broilers with NE infection. The effects of NE and tannic acid on dynamic metabolites of the intestinal flora (e.g., short-chain fatty acids) need further investigation.

The intestine is an effective barrier against pathogenic bacterial infection [43]. The intestinal mucosa and tight junctions are disrupted during intestinal inflammation, thereby increasing intestinal permeability [44,45]. In the present study, serum D-LA and DAO concentrations were higher in broiler chickens with NE, suggesting that NE impairs intestinal barrier integrity. This is consistent with the results of a previous study [23,46,47]. The decline in serum D-LA and DAO concentrations observed in broilers after the addition of tannic acid, indicating the potential positive impact of tannic acid on maintaining intestinal barrier integrity in broilers with NE. Similarly, a recent study demonstrated that the inclusion of chestnut tannins in the diet significantly reduced serum DAO concentrations in broilers subjected to heat stress [48]. Liu et al. found that dietary addition of different tannins could increase gene expression of *zonula occludens-1*, *Claudin-1*, and *Occludin* in broiler jejunum [31]. Yu et al. revealed that the addition of tannic acid to the diet of weaned piglets increased the mRNA expression concentrations of *Occludin* and *zonula occludens-1*, leading to enhanced intestinal barrier function and reduced concentrations of serum DAO and D-LA [49]. These studies have demonstrated the positive effects of tannic acid on intestinal barrier function; however, the specific mechanisms need to be further investigated.

In order to investigate the impact of tannic acid on immunity in broiler chickens with NE, we assessed the expression of relevant pro- and anti-inflammatory factors in both the intestine and serum. Proinflammatory and anti-inflammatory cytokines interact with each other, and their dynamic balance plays a crucial role in the development and outcome of inflammation. Previous studies have demonstrated that NE can induce inflammation in the intestine and the whole organism, leading to impaired intestinal health and reduced productivity [50,51,52,53,54]. Consistent with these findings, our study revealed that NE triggered inflammatory responses in both the intestine and the organism. Following NE infection in broiler chickens, the proinflammatory factor TNF-α increased and the anti-inflammatory factors IL-4 and IL-10 decreased, disrupting the dynamic balance and promoting inflammation. However, we observed that the addition of tannins decreased the concentrations of proinflammatory factors and increased the concentrations of anti-inflammatory factors, suggesting a potential anti-inflammatory effect of tannins. In support of this, Park et al. demonstrated that condensed tannins extracted from blackberry seeds inhibited nitric oxide production in lipopolysaccharide-induced macrophages [55]. Liu et al. reported that hydrolyzed tannins could reduce ear swelling and inflammatory responses in mouse models of arthritis [56]. Peng et al. demonstrated that tannin improved the immune function of broilers and inhibited liver inflammation by blocking the TLR4/NF-κB pathway [57]. Furthermore, our previous study indicated that the addition of tannins reduced the concentrations of MPO and CRP in serum [58]. The immunomodulatory effects of tannins may be attributed to their complex structure. Additionally, our previous research indicates that tannic acid may influence the immune response in the intestine by inhibiting the concentrations of *Clostridium perfringens*, modulating the intestinal microbiota, and reducing intestinal pathogen-induced stimulation [58]. The protective effects of tannins after NE infection in broiler chickens may be associated with the enhancement of anti-inflammatory function; however, the exact underlying mechanism requires further investigation.

Complex microorganisms in the gut play a key role in nutrition and intestinal health. NE is an intestinal disease that can disrupt intestinal microbial homeostasis and lead to intestinal ecological dysbiosis. To elucidate the mechanism by which tannic acid improves intestinal health, we further analyzed the microbes in the cecum. We observed that cecum microbial α-diversity was significantly decreased and β-diversity was significantly altered after NE infection compared with the NC group. These results are consistent with those of previous studies [59,60,61], and indicate that NE significantly alters the gut microbiota. In our experiment, the relative abundance of Clostridia as well as *Clostridium* sp. in the cecum flora of NE broilers was elevated, which may be caused by NE-inducing changes in the gut microbiota [60]. Although the relative abundance of Clostridia was elevated, due to the limitations of the 16S assay we could not determine whether this was *C. perfringens* CVCC52. In our previous study [58] we quantified the abundance of *C. perfringens* in the cecum and found that its abundance was significantly elevated in NE-infected broilers. In addition, *Oscillospira* is a dominant genus in the PC group, which was observed by Tang et al. as well [21]. The increased abundance of *Oscillospira* is possibly associated with intestinal inflammation [62]; its increased abundance has additionally been positively correlated with reduced body weight [63,64]. *Streptococcus* is a major strain of colorectal cancer [65]; we found that its abundance was significantly increased in broilers with NE. Therefore, NE is likely to elevate the relative abundance of Clostridia, *Streptococcus*, and *Oscillospira* in the cecal microbiota of broiler chickens. These findings indicate the significance of these bacteria in contributing to the adverse consequences associated with NE. In contrast, the NC group exhibited a predominance of beneficial bacteria such as *Lactococcus* and Rikenella, which are known for their production of short-chain fatty acids, in comparison to the PC group [66]. *Ruminococcaceae* are part of the natural intestinal flora of chickens; they helps with sugar absorption by cells, produce short-chain fatty acids, and inhibit the growth of *C. perfringens* bacteriocins [67], which is beneficial in maintaining intestinal health and may lead to weight gain [68,69,70]. Studies have reported that abundance of *Faecalibacterium* is reduced in the intestinal flora of broiler chickens with coccidial infection or heat stress [71,72]. *Faecalibacterium* can produce butyrate, which is associated with the maintenance of intestinal health [73]. *Bacteroides_dorei* reduces lipopolysaccharide production in the intestine and effectively inhibits the proinflammatory response [74]; simultaneously, it can promote the proliferation of *Lactobacillus* and *Bifidobacterium* [75]. Our study suggests that the increased abundance of *Ruminococcaceae*, *Faecalibacterium*, and *Bacteroides_dorei* following the addition of tannic acid is a contributing factor to the observed improvement intestinal health. Our findings indicate that NE disrupts the balance of intestinal flora and that the enhancement of beneficial bacterial growth associated with tannic acid supplementation may contribute to the amelioration of NE symptoms and restoration of intestinal health.

## 5. Conclusions

In the present study, we found that NE caused oxidative stress in broilers and disrupted their intestinal flora, thereby disrupting the barrier function of the intestine. Addition of tannic acid to the diet could mitigate the negative effects caused by NE by improving antioxidant capacity and anti-inflammatory capacity, regulating intestinal microbiota, and reducing intestinal permeability. 

Among the different dosages tested, the administration of 1000 mg/kg tannic acid exhibited enhanced antioxidant capacity in broiler chickens. Furthermore, tannic acid at doses ranging from 500 to 1000 mg/kg demonstrated improved anti-inflammatory capacity. A dosage of 500–750 mg/kg tannic acid was effective in reducing intestinal barrier permeability. However, further studies and investigation of the effects of different structures and doses of tannic acid on intestinal health, immunity, and antioxidant function and their mechanisms are warranted.

## Figures and Tables

**Table 1:** Ingredients and nutrient content of the chicken feed used during the experiment.

	Starter (d 1–14, %)	Grower (d 15–28, %)	Finisher (d 29–35, %)
Ingredients			
Corn	54.12	58.00	61.79
Soybean meal	32.45	28.00	25.68
Corn gluten meal	5.00	4.00	2.50
Soybean oil	3.00	4.62	4.50
Flour	0.90	0.90	1.05
Calcium hydrogen phosphate	2.00	1.95	1.85
Stone powder	1.00	1.00	1.07
Sodium chloride	0.30	0.30	0.30
L-lysine hydrochloride, 78%	0.30	0.30	0.36
DL-Methionine, 98%	0.25	0.25	0.19
Threonine	0.10	0.10	0.10
Arginine	0.04	0.04	0.06
Choline chloride, 50%	0.20	0.20	0.20
Broilers—Mineral premix ^1^	0.20	0.20	0.20
Broilers—Vitamin premix ^2^	0.03	0.03	0.03
Phytase 10,000	0.01	0.01	0.02
Zeolite	0.10	0.10	0.10
Total	100.00	100.00	100.00
Nutrient content ^3^			
Metabolic energy, Mcal/kg	2.99	3.10	3.10
Crude protein, %	22.39	20.12	18.5
Lysine, %	1.29	1.17	1.14
Methionine, %	0.61	0.57	0.48
Cystine, %	0.93	0.86	0.75
Threonine, %	0.92	0.83	0.77
Calcium, %	1.08	1.05	1.03
Available phosphorus, %	0.44	0.42	0.40

^1^ per kg of trace element premixed feed: copper, 8 g; iron, 40 g; zinc, 55 g; manganese, 60 g; iodine, 750 mg; selenium, 150 mg; and cobalt, 250 mg. ^2^ per kg of vitamin premix feed: vitamin A, 50 million IU; vitamin D3, 12 million IU; vitamin E, 100,000 IU; vitamin K3, 10 g; vitamin B1, 8 g; vitamin B2, 32 g; vitamin B6, 12 g; vitamin B12, 100 mg; niacin, 150 g; D-pantothenic acid, 46 g; folic acid, 5 g; biotin, 500 mg. ^3^ Calculated values based on the experimental diet analysis.

**Table 2:** Effects of tannic acid addition on the fecal water content of broiler chickens with necrotic enteritis (%).

	NC	PC	PTA1	PTA2	PTA3	PTA4	*p*-Value ^1^	Linear ^2^	Quadratic ^2^
D 25	70.36 ± 0.35	71.94 ± 0.81	70.16 ± 0.44	70.32 ± 0.33	70.19 ± 0.33	70.04 ± 0.50	0.083	0.027	0.133
D 26	72.72 ± 0.21 ^a^	73.07 ± 0.57 ^a^	71.51 ± 0.45 ^b^	71.41 ± 0.35 ^b^	71.11 ± 0.19 ^b^	71.10 ± 0.32 ^b^	0.001	0.002	0.060
D 27	71.60 ± 0.36	72.24 ± 0.45	71.49 ± 0.38	71.05 ± 0.22	71.35 ± 0.07	71.28 ± 0.20	0.149	0.035	0.070
D 28	71.74 ± 0.45 ^b^	73.92 ± 0.25 ^a^	73.86 ± 0.34 ^a^	73.32 ± 0.28 ^a^	73.62 ± 0.18 ^a^	73.53 ± 0.09 ^a^	<0.001	0.194	0.394
D 25–28	71.61 ± 0.22 ^b^	72.79 ± 0.43 ^a^	71.76 ± 0.30 ^b^	71.53 ± 0.20 ^b^	71.57 ± 0.11 ^b^	71.49 ± 0.24 ^b^	0.011	0.003	0.043
D 35	71.09 ± 0.32 ^b^	73.29 ± 0.63 ^a^	71.72 ± 0.49 ^b^	71.90 ± 0.61 ^b^	71.12 ± 0.20 ^b^	71.19 ± 0.25 ^b^	0.011	0.003	0.200

^1^ Overall *p*-values obtained from ANOVA; ^2^ *p*-values obtained using contrast trend analysis. Different lower-case letters in the same column indicate significant differences (*p* < 0.05). Data are the mean ± SEM. *n =* 7. NC: unchallenged and untreated; PC: NE challenged and untreated; PTA1: NE challenged and control diet supplemented with 250 mg/kg tannic acid; PTA2: NE challenged and control diet supplemented with 500 mg/kg tannic acid; PTA3: NE challenged and control diet supplemented with 750 mg/kg tannic acid; PTA4: NE challenged and control diet supplemented with 1000 mg/kg tannic acid.

**Table 3:** Effects of tannic acid addition on the pH of intestinal chyme of broiler chickens with necrotic enteritis.

	NC	PC	PTA1	PTA2	PTA3	PTA4	*p*-Value ^1^	Linear ^2^	Quadratic ^2^
D 28									
Chyme pH of Ileum	7.31 ± 0.09 ^a^	5.18 ± 0.14 ^b^	5.49 ± 0.28 ^b^	5.54 ± 0.12 ^b^	5.59 ± 0.14 ^b^	5.40 ± 0.13 ^b^	<0.001	0.337	0.125
Chyme pH of Cecum	7.23 ± 0.08 ^a^	6.73 ± 0.16 ^b^	6.87 ± 0.12 ^b^	6.57 ± 0.06 ^b^	6.88 ± 0.10 ^b^	6.88 ± 0.25 ^b^	0.002	0.542	0.557
D 35									
Chyme pH of Ileum	6.82 ± 0.16	6.16 ± 0.27	6.27 ± 0.08	6.13 ± 0.12	5.91 ± 0.11	5.77 ± 0.21	0.057	0.043	0.373
Chyme pH of Cecum	6.96 ± 0.05	6.92 ± 0.05	6.91 ± 0.08	6.87 ± 0.06	6.76 ± 0.11	6.78 ± 0.07	0.302	0.081	0.969

^1^ Overall *p*-values obtained from ANOVA; ^2^ *p*-values obtained using contrast trend analysis. Different lower-case letters in the same column indicate significant differences (*p* < 0.05). Data are the mean ± SEM. *n =* 7. NC: unchallenged and untreated; PC: NE challenged and untreated; PTA1: NE challenged and control diet supplemented with 250 mg/kg tannic acid; PTA2: NE challenged and control diet supplemented with 500 mg/kg tannic acid; PTA3: NE challenged and control diet supplemented with 750 mg/kg tannic acid; PTA4: NE challenged and control diet supplemented with 1000 mg/kg tannic acid.

**Table 4:** Effects of tannic acid addition on the physical barrier function of the intestine in broiler chickens with necrotic enteritis.

	NC	PC	PTA1	PTA2	PTA3	PTA4	*p*-Value ^1^	Linear ^2^	Quadratic ^2^
D 28									
DAO, U/L	21.13 ± 2.51	28.40 ± 2.20	20.94 ± 2.41	19.38 ± 3.53	20.58 ± 0.95	22.42 ± 1.78	0.127	0.105	0.020
D-LA, mmol/L	0.51 ± 0.07 ^b^	0.82 ± 0.04 ^a^	0.63 ± 0.10 ^ab^	0.53 ± 0.08 ^b^	0.59 ± 0.06 ^b^	0.84 ± 0.08 ^a^	0.006	0.997	0.001
D 35									
DAO, U/L	20.60 ± 1.17 ^ab^	28.14 ±5.30 ^a^	21.15 ± 3.88 ^ab^	13.72 ± 1.82 ^b^	17.46 ± 3.57 ^b^	15.61 ± 1.74 ^b^	0.050	0.015	0.115
D-LA, mmol/L	0.86 ± 0.07	0.90 ± 0.07	0.95 ± 0.13	0.95 ± 0.10	0.86 ± 0.04	0.88 ± 0.06	0.913	0.614	0.597

^1^ Overall *p*-values obtained from ANOVA; ^2^ *p*-values obtained using contrast trend analysis. Different lower-case letters in the same column indicate significant differences (*p* < 0.05). Data are the mean ± SEM. *n =* 7. NC: unchallenged and untreated; PC: NE challenged and untreated; PTA1: NE challenged and control diet supplemented with 250 mg/kg tannic acid; PTA2: NE challenged and control diet supplemented with 500 mg/kg tannic acid; PTA3: NE challenged and control diet supplemented with 750 mg/kg tannic acid; PTA4: NE challenged and control diet supplemented with 1000 mg/kg tannic acid. DAO: diamine oxidase; D-LA: D-lactic acid.

**Table 5:** Effects of tannic acid on the cytokine contents of broiler chickens infected with necrotic enteritis.

	NC	PC	PTA1	PTA2	PTA3	PTA4	*p*-Value ^1^	Linear ^2^	Quadratic ^2^
Serum									
D 28									
TNF-α, pg/mL	31.64 ± 2.94 ^d^	59.65 ± 3.68 ^a^	54.10 ± 2.37 ^ab^	47.53 ± 1.50 ^b^	39.67 ± 1.23 ^c^	48.02 ± 2.90 ^b^	<0.001	<0.001	0.008
IL-10, pg/mL	70.24 ± 2.77 ^a^	45.65 ± 1.49 ^c^	53.55 ± 3.78 ^b^	53.95 ± 3.33 ^b^	61.80 ± 1.63 ^b^	60.28 ± 2.35 ^b^	<0.001	<0.001	0.264
IL-4, pg/mL	139.76 ± 4.43 ^a^	99.85 ± 4.60 ^c^	111.06 ± 4.89 ^bc^	124.74 ± 7.01 ^ab^	135.51 ± 8.07 ^a^	121.84 ± 7.69 ^ab^	0.001	0.003	0.041
D 35									
TNF-α, pg/mL	36.25 ± 2.73 ^d^	60.11 ± 1.42 ^a^	53.03 ± 2.53 ^ab^	45.75 ± 3.23 ^bc^	43.96 ± 3.51 ^c^	44.20 ± 1.65 ^c^	<0.001	<0.001	0.048
IL-10, pg/mL	74.03 ± 3.68 ^a^	37.85 ± 3.24 ^d^	60.89 ± 2.44 ^bc^	57.97 ± 3.57 ^c^	68.85 ± 3.34 ^ab^	58.19 ± 2.23 ^c^	<0.001	<0.001	<0.001
IL-4, pg/mL	156.64 ± 7.67 ^a^	73.39 ± 10.23 ^d^	116.12 ± 9.12 ^c^	128.15 ± 7.40 ^bc^	140.43 ± 3.87 ^ab^	117.46 ± 3.79 ^c^	<0.001	<0.001	<0.001
Ileal									
D 28									
TNF-α, pg/mg.pro	11.39 ± 1.08 ^b^	25.96 ± 3.04 ^a^	17.30 ± 0.92 ^b^	25.54 ± 3.44 ^a^	14.92 ± 1.24 ^b^	15.72 ± 1.46 ^b^	0.001	0.003	0.994
IL-10, pg/mg.pro	32.16 ± 2.63 ^a^	18.78 ± 0.83 ^bc^	16.33 ± 0.44 ^c^	25.09 ± 3.07 ^b^	24.92 ± 2.06 ^b^	21.64 ± 1.87 ^bc^	<0.001	0.024	0.148
IL-4, pg/mg.pro	68.11 ± 4.96 ^a^	35.71 ± 2.10 ^cd^	29.14 ± 1.94 ^d^	55.60 ± 7.20 ^b^	46.45 ± 3.75 ^bc^	47.31 ± 2.36 ^bc^	<0.001	0.003	0.175
D 35									
TNF-α, pg/mg.pro	11.05 ± 1.10 ^c^	23.83 ± 2.19 ^a^	26.50 ± 2.18 ^a^	17.41 ± 1.25 ^b^	17.71 ± 2.01 ^b^	15.75 ± 1.76 ^bc^	<0.001	<0.001	0.986
IL-10, pg/mg.pro	31.18 ± 3.06 ^a^	10.44 ± 0.52 ^c^	24.00 ± 2.41 ^b^	24.04 ± 1.41 ^b^	24.64 ± 1.93 ^b^	23.53 ± 1.67 ^b^	<0.001	<0.001	<0.001
IL-4, pg/mg.pro	63.44 ± 9.26 ^a^	25.50 ± 3.35 ^c^	51.47 ± 3.24 ^ab^	51.89 ± 4.88 ^ab^	44.48 ± 2.97 ^b^	49.71 ± 2.00 ^ab^	<0.001	0.001	0.001

^1^ Overall *p*-values obtained from ANOVA; ^2^ *p*-values obtained using contrast trend analysis. Different lower-case letters in the same column indicate significant differences (*p* < 0.05). Data are the mean ± SEM. *n =* 7. NC: unchallenged and untreated; PC: NE challenged and untreated; PTA1: NE challenged and control diet supplemented with 250 mg/kg tannic acid; PTA2: NE challenged and control diet supplemented with 500 mg/kg tannic acid; PTA3: NE challenged and control diet supplemented with 750 mg/kg tannic acid; PTA4: NE challenged and control diet supplemented with 1000 mg/kg tannic acid. TNF-α: Tumor necrosis factor-α; IL-10: Interleukin-10; IL-4: Interleukin-4.

**Table 6:** Effects of tannic acid addition on the antioxidant function of the serum and ileal mucosa in broiler chickens with necrotic enteritis.

	NC	PC	PTA1	PTA2	PTA3	PTA4	*p*-Value ^1^	Linear ^2^	Quadratic ^2^
Serum									
D 28									
T-AOC, Mm	4.04 ± 0.07 ^ab^	3.66 ± 0.29 ^b^	3.45 ± 0.27 ^b^	3.34 ± 0.40 ^b^	4.27 ± 0.44 ^ab^	4.98 ± 0.29 ^a^	0.008	0.003	0.033
SOD, U/mL	27,594 ± 627	24,362 ± 3123	26,447 ± 1736	26,038 ± 2943	29,234 ± 893	25,250 ± 2989	0.722	0.568	0.368
MDA, nmol/mL	2.14 ± 0.13 ^b^	3.11 ± 0.23 ^a^	3.21 ± 0.32 ^a^	2.82 ± 0.16 ^a^	2.79 ± 0.05 ^a^	2.77 ± 0.14 ^a^	0.007	0.091	0.882
D 35									
T-AOC, Mm	5.02 ± 0.22	4.62 ± 0.15	4.93 ± 0.10	5.22 ± 0.12	4.97 ± 0.12	5.08 ± 0.09	0.093	0.016	0.041
SOD, U/mL	24,636 ± 782	26,792 ± 579	29,346± 3381	28,992 ± 611	28,266 ± 1930	27,555± 2038	0.514	0.944	0.364
MDA, nmol/mL	2.44 ± 0.33	3.35 ± 0.39	3.07 ± 0.22	2.61 ± 0.15	2.83 ± 0.09	2.79 ± 0.19	0.162	0.074	0.195
Ileal mucosa									
D 28									
T-AOC, Mm/mg prot	0.26 ± 0.01 ^a^	0.10 ± 0.02 ^b^	0.09 ± 0.01 ^b^	0.10 ± 0.02 ^b^	0.13 ± 0.01 ^b^	0.13 ± 0.01 ^b^	<0.001	0.032	0.725
SOD, U/mg prot	5892 ± 508	6055 ± 411	5928± 524	6274 ± 843	6562 ± 721	6157 ± 831	0.981	0.703	0.813
MDA, nomL/mg prot	2.12 ± 0.71	2.44 ± 0.41	2.34 ± 0.21	2.24 ± 0.22	1.47 ± 0.13	2.66 ± 0.16	0.199	0.578	0.047
D 35									
T-AOC, Mm/mg prot	0.16 ± 0.03	0.10 ± 0.02	0.10 ± 0.03	0.12 ± 0.02	0.16 ± 0.04	0.18 ± 0.03	0.217	0.020	0.668
SOD, U/mg prot	7998 ± 223	9702 ± 582	11,081 ± 910	10,035 ± 827	10,965 ± 1252	9403 ± 387	0.083	0.791	0.227
MDA, nomL/mg prot	1.74 ± 0.57	3.11 ± 0.25	2.24 ± 0.38	1.63 ± 0.79	1.83 ± 0.38	2.67 ± 0.60	0.140	0.323	0.010

^1^ Overall *p*-values obtained from ANOVA; ^2^ *p*-values obtained using contrast trend analysis. Different lower-case letters in the same column indicate significant differences (*p* < 0.05). Data are the mean ± SEM. *n =* 7. NC: unchallenged and untreated; PC: NE challenged and untreated; PTA1: NE challenged and control diet supplemented with 250 mg/kg tannic acid; PTA2: NE challenged and control diet supplemented with 500 mg/kg tannic acid; PTA3: NE challenged and control diet supplemented with 750 mg/kg tannic acid; PTA4: NE challenged and control diet supplemented with 1000 mg/kg tannic acid. MDA: malondialdehyde; SOD: superoxide dismutase; T-AOC: total antioxidant capacity.

**Table 7:** ANOSIM method to compare similarities in bacterial flora composition between the three treatments.

Treatments	R Values ^1^	*p* Values
D 28		
NC-PC	0.261	0.010
PC-PTA3	0.381	0.001
D 35		
NC-PC	0.113	0.036
PC-PTA3	0.034	0.089

^1^ R values range from −1 to 1. Differences between groups are significant for R values > 0 and differences within groups are greater than differences between groups for R values < 0. *p* < 0.05 indicates significant differences. *n* = 7.

## Data Availability

All data generated or analyzed during this study are available from the corresponding author upon request.

## Abbreviations

DAO: diamine oxidase: D-LA: D-lactic acid; MDA: malondialdehyde; NE: necrotic enteritis; SOD: superoxide dismutase; T-AOC: total antioxidant capacity; TNF-α: Tumor necrosis factor-α; IL-10: Interleukin-10; IL-4: Interleukin-4.

## References

[1] Smedley J.G., Fisher D.J., Sayeed S., Chakrabarti G., McClane B.A. (2004). The enteric toxins of Clostridium perfringens. Rev. Physiol. Biochem. Pharmacol..

[2] Engström B.E., Fermér C., Lindberg A., Saarinen E., Båverud V., Gunnarsson A. (2003). Molecular typing of isolates of Clostridium perfringens from healthy and diseased poultry. Vet. Microbiol..

[3] Wade B., Keyburn A.J.W.P. (2015). The true cost of necrotic enteritis. Poult. World.

[4] Emami N.K., Dalloul R.A. (2021). Centennial Review: Recent developments in host-pathogen interactions during necrotic enteritis in poultry. Poult. Sci..

[5] Huyghebaert G., Ducatelle R., Van Immerseel F. (2011). An update on alternatives to antimicrobial growth promoters for broilers. Vet. J..

[6] Huang Q., Liu X., Zhao G., Hu T., Wang Y. (2018). Potential and challenges of tannins as an alternative to in-feed antibiotics for farm animal production. Anim. Nutr..

[7] Schiavone A., Guo K., Tassone S., Gasco L., Hernandez E., Denti R., Zoccarato I. (2008). Effects of a natural extract of chestnut wood on digestibility, performance traits, and nitrogen balance of broiler chicks. Poult. Sci..

[8] Redondo L.M., Chacana P.A., Dominguez J.E., Fernandez Miyakawa M.E. (2014). Perspectives in the use of tannins as alternative to antimicrobial growth promoter factors in poultry. Front. Microbiol..

[9] Jamroz D., Wiliczkiewicz A., Skorupińska J., Orda J., Kuryszko J., Tschirch H. (2009). Effect of sweet chestnut tannin (SCT) on the performance, microbial status of intestine and histological characteristics of intestine wall in chickens. Br. Poult. Sci..

[10] Choi J., Marshall B., Ko H., Shi H., Singh A.K., Thippareddi H., Holladay S., Gogal R.M., Kim W.K. (2022). Antimicrobial and immunomodulatory effects of tannic acid supplementation in broilers infected with Salmonella Typhimurium. Poult. Sci..

[11] Redondo L.M., Dominguez J.E., Rabinovitz B.C., Redondo E.A., Fernández Miyakawa M.E. (2015). Hydrolyzable and condensed tannins resistance in Clostridium perfringens. Anaerobe.

[12] Elizondo A.M., Mercado E.C., Rabinovitz B.C., Fernandez-Miyakawa M.E. (2010). Effect of tannins on the in vitro growth of Clostridium perfringens. Vet. Microbiol..

[13] Yitbarek A., Echeverry H., Brady J., Hernandez-Doria J., Camelo-Jaimes G., Sharif S., Guenter W., House J.D., Rodriguez-Lecompte J.C. (2012). Innate immune response to yeast-derived carbohydrates in broiler chickens fed organic diets and challenged with Clostridium perfringens. Poult. Sci..

[14] Du E., Wang W., Gan L., Li Z., Guo S., Guo Y. (2016). Effects of thymol and carvacrol supplementation on intestinal integrity and immune responses of broiler chickens challenged with Clostridium perfringens. J. Anim. Sci. Biotechnol..

[15] Fasina Y.O., Lillehoj H.S. (2019). Characterization of intestinal immune response to Clostridium perfringens infection in broiler chickens. Poult. Sci..

[16] Wu Y., Shao Y., Song B., Zhen W., Wang Z., Guo Y., Shahid M.S., Nie W. (2018). Effects of Bacillus coagulans supplementation on the growth performance and gut health of broiler chickens with Clostridium perfringens-induced necrotic enteritis. J. Anim. Sci. Biotechnol..

[17] Burnell T.W., Cromwell G.L., Stahly T.S. (1988). Effects of dried whey and copper sulfate on the growth responses to organic acid in diets for weanling pigs. J. Anim. Sci..

[18] Zhang B., Lv Z., Li Z., Wang W., Li G., Guo Y. (2018). Dietary l-arginine Supplementation Alleviates the Intestinal Injury and Modulates the Gut Microbiota in Broiler Chickens Challenged by Clostridium perfringens. Front. Microbiol..

[19] Zhao Y., Zeng D., Wang H., Qing X., Sun N., Xin J., Luo M., Khalique A., Pan K., Shu G. (2020). Dietary probiotic bacillus licheniformis h2 enhanced growth performance, morphology of small intestine and liver, and antioxidant capacity of broiler chickens against clostridium perfringens-induced subclinical necrotic enteritis. Probiotics Antimicrob. Proteins.

[20] Sun Y., Ni A., Jiang Y., Li Y., Huang Z., Shi L., Xu H., Chen C., Li D., Han Y. (2020). Effects of replacing in-feed antibiotics with synergistic organic acids on growth performance, health, carcass, and immune and oxidative statuses of broiler chickens under clostridium perfringens type a challenge. Avian Dis..

[21] Tang Y., Zhang X., Wang Y., Guo Y., Zhu P., Li G., Zhang J., Ma Q., Zhao L. (2022). Dietary ellagic acid ameliorated Clostridium perfringens-induced subclinical necrotic enteritis in broilers via regulating inflammation and cecal microbiota. J. Anim. Sci. Biotechnol..

[22] Zhou M., Zeng D., Ni X., Tu T., Yin Z., Pan K., Jing B. (2016). Effects of Bacillus licheniformis on the growth performance and expression of lipid metabolism-related genes in broiler chickens challenged with Clostridium perfringens-induced necrotic enteritis. Lipids Health Dis..

[23] Li P., Liu C., Niu J., Zhang Y., Li C., Zhang Z., Guo S., Ding B. (2022). Effects of Dietary Supplementation with Vitamin A on Antioxidant and Intestinal Barrier Function of Broilers Co-Infected with Coccidia and Clostridium perfringens. Animals.

[24] El-Demerdash A.S., Mohamady S.N., Megahed H.M., Ali N.M. (2023). Evaluation of gene expression related to immunity, apoptosis, and gut integrity that underlies Artemisia’s therapeutic effects in necrotic enteritis-challenged broilers. 3 Biotech.

[25] Tian T., Wang Z., Zhang J. (2017). Pathomechanisms of oxidative stress in inflammatory bowel disease and potential antioxidant therapies. Oxid. Med. Cell. Longev..

[26] Li S., Xu M., Niu Q., Xu S., Ding Y., Yan Y., Guo S., Li F. (2015). Efficacy of procyanidins against in vivo cellular oxidative damage: A systematic review and meta-analysis. PLoS ONE.

[27] Smeriglio A., Barreca D., Bellocco E., Trombetta D. (2017). Proanthocyanidins and hydrolysable tannins: Occurrence, dietary intake and pharmacological effects. Br. J. Pharmacol..

[28] Wang M., Huang H., Liu S., Zhuang Y., Yang H., Li Y., Chen S., Wang L., Yin L., Yao Y. (2019). Tannic acid modulates intestinal barrier functions associated with intestinal morphology, antioxidative activity, and intestinal tight junction in a diquat-induced mouse model. RSC Adv..

[29] Azimullah S., Meeran M.F.N., Ayoob K., Arunachalam S., Ojha S., Beiram R. (2023). Tannic Acid Mitigates Rotenone-Induced Dopaminergic Neurodegeneration by Inhibiting Inflammation, Oxidative Stress, Apoptosis, and Glutamate Toxicity in Rats. Int. J. Mol. Sci..

[30] Kizir D., Karaman M., Ceylan H. (2023). Tannic acid may ameliorate doxorubicin-induced changes in oxidative stress parameters in rat spleen. Naunyn-Schmiedeberg’s Arch. Pharmacol..

[31] Liu S., Wang K., Lin S., Zhang Z., Cheng M., Hu S., Hu H., Xiang J., Chen F., Li G. (2023). Comparison of the Effects between Tannins Extracted from Different Natural Plants on Growth Performance, Antioxidant Capacity, Immunity, and Intestinal Flora of Broiler Chickens. Antioxidants.

[32] Sieniawska E. (2015). Activities of tannins--from in vitro studies to clinical trials. Nat. Prod. Commun..

[33] Liang W., Li H., Zhou H., Wang M., Zhao X., Sun X., Li C., Zhang X. (2021). Effects of Taraxacum and Astragalus extracts combined with probiotic Bacillus subtilis and Lactobacillus on Escherichia coli-infected broiler chickens. Poult. Sci..

[34] Lee J.H., Lee B., Rousseau X., Gomes G.A., Oh H.J., Kim Y.J., Chang S.Y., An J.W., Go Y.B., Song D.C. (2022). Stimbiotic supplementation modulated intestinal inflammatory response and improved boilers performance in an experimentally-induced necrotic enteritis infection model. J. Anim. Sci. Biotechnol..

[35] Yang Y., Luo H., Song X., Yu L., Xie J., Yang J., Jia R., Lin J., Zou Y., Li L. (2017). Preparation of galla chinensis oral solution as well as its stability, safety, and antidiarrheal activity evaluation. Evid. Based Complement. Alternat. Med..

[36] Girard M., Thanner S., Pradervand N., Hu D., Ollagnier C., Bee G. (2018). Hydrolysable chestnut tannins for reduction of postweaning diarrhea: Efficacy on an experimental ETEC F4 model. PLoS ONE.

[37] Choi J., Tompkins Y.H., Teng P.Y., Gogal R.M., Kim W.K. (2022). Effects of tannic acid supplementation on growth performance, oocyst shedding, and gut health of in broilers infected with eimeria maxima. Animals.

[38] Kim T.J., Silva J.L., Kim M.K., Jung Y.S. (2010). Enhanced antioxidant capacity and antimicrobial activity of tannic acid by thermal processing. Food Chem..

[39] Sivasankar C., Jha N.K., Ghosh R., Shetty P.H. (2020). Anti quorum sensing and anti virulence activity of tannic acid and it’s potential to breach resistance in Salmonella enterica Typhi / Paratyphi A clinical isolates. Microb. Pathog..

[40] Tsiouris V., Georgopoulou I., Batzios C., Pappaioannou N., Ducatelle R., Fortomaris P. (2018). Heat stress as a predisposing factor for necrotic enteritis in broiler chicks. Avian Pathol..

[41] Wu S.B., Rodgers N.J., Cui G., Sun Y., Choct M. (2016). Dynamics of intestinal metabolites and morphology in response to necrotic enteritis challenge in broiler chickens. Avian Pathol..

[42] Gharib-Naseri K., Kheravii S.K., Keerqin C., Morgan N., Swick R.A., Choct M., Wu S.B. (2019). Two different Clostridium perfringens strains produce different levels of necrotic enteritis in broiler chickens. Poult. Sci..

[43] Sánchez de Medina F., Romero-Calvo I., Mascaraque C., Martínez-Augustin O. (2014). Intestinal inflammation and mucosal barrier function. Inflamm. Bowel Dis..

[44] Vicuña E.A., Kuttappan V.A., Tellez G., Hernandez-Velasco X., Seeber-Galarza R., Latorre J.D., Faulkner O.B., Wolfenden A.D., Hargis B.M., Bielke L.R. (2015). Dose titration of FITC-D for optimal measurement of enteric inflammation in broiler chicks. Poult. Sci..

[45] Barekatain R., Nattrass G., Tilbrook A.J., Chousalkar K., Gilani S. (2019). Reduced protein diet and amino acid concentration alter intestinal barrier function and performance of broiler chickens with or without synthetic glucocorticoid. Poult. Sci..

[46] Kumar A., Toghyani M., Kheravii S.K., Pineda L., Han Y., Swick R.A., Wu S.B. (2022). Organic acid blends improve intestinal integrity, modulate short-chain fatty acids profiles and alter microbiota of broilers under necrotic enteritis challenge. Anim. Nutr..

[47] Zhang R., Qin S., Yang C., Niu Y., Feng J. (2023). The protective effects of Bacillus licheniformis against inflammatory responses and intestinal barrier damage in broilers with necrotic enteritis induced by Clostridium perfringens. J. Sci. Food Agric..

[48] Liu H.W., Li K., Zhao J.S., Deng W. (2018). Effects of chestnut tannins on intestinal morphology, barrier function, pro-inflammatory cytokine expression, microflora and antioxidant capacity in heat-stressed broilers. J. Anim. Physiol. Anim. Nutr..

[49] Yu J., Song Y., Yu B., He J., Zheng P., Mao X., Huang Z., Luo Y., Luo J., Yan H. (2020). Tannic acid prevents post-weaning diarrhea by improving intestinal barrier integrity and function in weaned piglets. J. Anim. Sci. Biotechnol..

[50] Song B., Li P., Yan S., Liu Y., Gao M., Lv H., Lv Z., Guo Y. (2022). Effects of Dietary Astragalus Polysaccharide Supplementation on the Th17/Treg Balance and the Gut Microbiota of Broiler Chickens Challenged with Necrotic Enteritis. Front. Immunol..

[51] Daneshmand A., Kermanshahi H., Mohammed J., Sekhavati M.H., Javadmanesh A., Ahmadian M., Alizadeh M., Razmyar J., Kulkarni R.R. (2022). Intestinal changes and immune responses during Clostridium perfringens-induced necrotic enteritis in broiler chickens. Poult. Sci..

[52] Lee Y.S., Lee S.H., Gadde U.D., Oh S.T., Lee S.J., Lillehoj H.S. (2018). Allium hookeri supplementation improves intestinal immune response against necrotic enteritis in young broiler chickens. Poult. Sci..

[53] Salem H.M., Ismael E., Shaalan M. (2021). Evaluation of the effects of silver nanoparticles against experimentally induced necrotic enteritis in broiler chickens. Int. J. Nanomed..

[54] Daneshmand A., Sharma N.K., Dao T.H., Barekatain R., Swick R.A., Wu S.B. (2023). Spray-dried porcine plasma enhances feed efficiency, intestinal integrity, and immune response of broilers challenged with necrotic enteritis. Poult. Sci..

[55] Park M., Cho H., Jung H., Lee H., Hwang K.T. (2014). Antioxidant and anti-inflammatory activities of tannin fraction of the extract from black raspberry seeds compared to grape seeds. J. Food Biochem..

[56] Liu J.B., Ding Y.S., Zhang Y., Chen J.B., Cui B.S., Bai J.Y., Lin M.B., Hou Q., Zhang P.C., Li S. (2015). Anti-inflammatory Hydrolyzable Tannins from Myricaria bracteata. J. Nat. Prod..

[57] Yuan P., Xu H., Ma Y., Niu J., Liu Y., Huang L., Jiang S., Jiao N., Yuan X., Yang W. (2023). Effects of dietary Galla Chinensis tannin supplementation on immune function and liver health in broiler chickens challenged with lipopolysaccharide. Front. Vet. Sci..

[58] Xu H., Fu J., Luo Y., Li P., Song B., Lv Z., Guo Y. (2023). Effects of tannic acid on the immunity and intestinal health of broiler chickens with necrotic enteritis infection. J. Anim. Sci. Biotechnol..

[59] Stanley D., Wu S.B., Rodgers N., Swick R.A., Moore R.J. (2014). Differential responses of cecal microbiota to fishmeal, Eimeria and Clostridium perfringens in a necrotic enteritis challenge model in chickens. PLoS ONE.

[60] Hernandez-Patlan D., Solis-Cruz B., Pontin K.P., Hernandez-Velasco X., Merino-Guzman R., Adhikari B., López-Arellano R., Kwon Y.M., Hargis B.M., Arreguin-Nava M.A. (2019). Impact of a Bacillus Direct-Fed Microbial on Growth Performance, Intestinal Barrier Integrity, Necrotic Enteritis Lesions, and Ileal Microbiota in Broiler Chickens Using a Laboratory Challenge Model. Front. Vet. Sci..

[61] Keerqin C., Rhayat L., Zhang Z.H., Gharib-Naseri K., Kheravii S.K., Devillard E., Crowley T.M., Wu S.B. (2021). Probiotic Bacillus subtilis 29,784 improved weight gain and enhanced gut health status of broilers under necrotic enteritis condition. Poult. Sci..

[62] Romano S., Savva G.M., Bedarf J.R., Charles I.G., Hildebrand F., Narbad A. (2021). Meta-analysis of the Parkinson’s disease gut microbiome suggests alterations linked to intestinal inflammation. NPJ Park. Dis..

[63] Konikoff T., Gophna U. (2016). Oscillospira: A central, enigmatic component of the human gut microbiota. Trends Microbiol..

[64] Chen Y.R., Zheng H.M., Zhang G.X., Chen F.L., Chen L.D., Yang Z.C. (2020). High Oscillospira abundance indicates constipation and low BMI in the Guangdong Gut Microbiome Project. Sci. Rep..

[65] Yang Y., Du L., Shi D., Kong C., Liu J., Liu G., Li X., Ma Y. (2021). Dysbiosis of human gut microbiome in young-onset colorectal cancer. Nat. Commun..

[66] Ma H., Hu Y., Zhang B., Shao Z., Wang S. (2022). Tea polyphenol—Gut microbiota interactions: Hints on improving the metabolic syndrome in a multi-element and multi-target manner. Food Sci. Hum. Wellness.

[67] Crost E.H., Ajandouz E.H., Villard C., Geraert P.A., Puigserver A., Fons M. (2011). Ruminococcin C, a new anti-Clostridium perfringens bacteriocin produced in the gut by the commensal bacterium Ruminococcus gnavus E1. Biochimie.

[68] Wang Y., Xu Y., Xu S., Yang J., Wang K., Zhan X. (2021). Bacillus subtilis DSM29784 alleviates negative effects on growth performance in broilers by improving the intestinal health under necrotic enteritis challenge. Front. Microbiol..

[69] Zhou Q., Lan F., Li X., Yan W., Sun C., Li J., Yang N., Wen C. (2021). The spatial and temporal characterization of gut microbiota in broilers. Front. Vet. Sci..

[70] Zhang J.M., Liu X.Y., Gu W., Xu H.Y., Jiao H.C., Zhao J.P., Wang X.J., Li H.F., Lin H. (2021). Different effects of probiotics and antibiotics on the composition of microbiota, SCFAs concentrations and FFAR2/3 mRNA expression in broiler chickens. J. Appl. Microbiol..

[71] Huang G., Tang X., Bi F., Hao Z., Han Z., Suo J., Zhang S., Wang S., Duan C., Yu Z. (2018). Eimeria tenella infection perturbs the chicken gut microbiota from the onset of oocyst shedding. Vet. Parasitol..

[72] Shi D., Bai L., Qu Q., Zhou S., Yang M., Guo S., Li Q., Liu C. (2019). Impact of gut microbiota structure in heat-stressed broilers. Poult. Sci..

[73] Borda-Molina D., Vital M., Sommerfeld V., Rodehutscord M., Camarinha-Silva A. (2016). Insights into broilers’ gut microbiota fed with phosphorus, calcium, and phytase supplemented diets. Front. Microbiol..

[74] Yoshida N., Emoto T., Yamashita T., Watanabe H., Hayashi T., Tabata T., Hoshi N., Hatano N., Ozawa G., Sasaki N. (2018). Bacteroides vulgatus and bacteroides dorei reduce gut microbial lipopolysaccharide production and inhibit atherosclerosis. Circulation.

[75] Gao G., Cao J., Mi L., Feng D., Deng Q., Sun X., Zhang H., Wang Q., Wang J. (2021). BdPUL12 depolymerizes β-mannan-like glycans into mannooligosaccharides and mannose, which serve as carbon sources for Bacteroides dorei and gut probiotics. Int. J. Biol. Macromol..

